# ‘Let’s work alongside it’: healthcare professionals’ perspectives of generative AI in supporting care at home in paediatric rheumatology

**DOI:** 10.1016/j.ero.2026.100258

**Published:** 2026-10-19

**Authors:** Klaudia H. Kupiec, Sabina Sweet, Sandrine Compeyrot-Lacassagne, Polly Livermore

**Affiliations:** 1 Department of Paediatric Rheumatology, Great Ormond Street Hospital National Health Service (NHS) Foundation Trust, London, UK; 2 University College London (UCL) Great Ormond Street Institute of Child Health, Infection, Immunity, and Inflammation Research and Teaching Department, University College London, London, UK; 3 National Institute for Health and Care Research (NIHR) Great Ormond Street Hospital Biomedical Research Centre, UCL Great Ormond Street Institute of Child Health, London, UK; 4 UCL Interaction Centre, Department of Computer Science and Division of Psychology and Language Sciences, University College London, London, UK

## Abstract

**Objectives:**

Generative artificial intelligence (GenAI) is increasingly explored in healthcare, yet its potential to support care at home remains largely unexamined. In paediatric rheumatology, where families frequently undertake complex tasks to manage treatment plans between appointments, safe and effective guidance is essential. The aim of the current study was to understand healthcare professionals’ (HCPs’) views on adopting GenAI tools to help families manage their child’s care.

**Methods:**

Utilising a vignette-based methodology, paediatric rheumatology HCPs were invited to focus groups and presented with 3 hypothetical scenarios: (1) injection administration, (2) flare assessment, and (3) physiotherapy exercises. Following the principles outlined by Braun and Clarke, data were analysed using thematic analysis.

**Results:**

A total of 37 HCPs from the wider multidisciplinary team from 14 different hospitals participated in this study. Six main themes were identified: (1) opportunities of GenAI to benefit patient care; (2) disadvantages and challenges; (3) risks, safety netting, and evaluation; (4) different approaches of utilising GenAI; (5) role of HCPs in the world of GenAI; and (6) ensuring inclusivity and accessibility.

**Conclusions:**

HCPs recognised opportunities for how GenAI could support caregivers in managing their child’s healthcare or young people’s self-management, particularly through guidance support rather than clinical interpretation. However, concerns were raised regarding the accuracy, reliability, privacy, and data governance of currently existing GenAI tools. The perspectives of HCPs are critical to ensuring that the application of GenAI in healthcare aligns with clinical expectations, ethical standards, regulatory processes, and the needs of patients and families.

**WHAT IS ALREADY KNOWN ON THIS TOPIC**

• Generative artificial intelligence (GenAI) is increasingly used in healthcare settings for diagnostic classifications, summarising healthcare records, interpreting imaging results, clinical reasoning, and decision-making.
• However, GenAI models face significant challenges, such as hallucinations and algorithmic bias, which may exacerbate disparities when underrepresented groups are insufficiently represented in training data.

**WHAT THIS STUDY ADDS**

• Healthcare professionals (HCPs) felt that, due to ongoing work pressures, utilising GenAI could be a valuable adjunct in supporting their capacity, with GenAI tools having the ability to function as an aid to complement care.
• HCPs cautioned that there are still several risks relating to GenAI in healthcare that cannot be ignored, such as privacy, safety, accuracy, as well as overuse and dependence.

**HOW THIS STUDY MIGHT AFFECT RESEARCH, PRACTICE OR POLICY**

• This study highlights the need for GenAI tools in healthcare to be carefully and responsibly monitored, appraised, and governed to overcome ongoing challenges and to ensure patient safety.
• This study further informs the need for codesign methodology for GenAI implementation and the development of standardised guidelines and regulations.

Alt-text: Unlabelled box dummy alt text

## INTRODUCTION

As generative artificial intelligence (GenAI) is increasingly used in healthcare settings, understanding the capabilities of this innovative technology is critical to ensure its safe and effective integration into healthcare. To facilitate this process, adoption and implementation strategies of GenAI should involve healthcare professionals’ expertise to maximise the quality of care. The term GenAI refers to systems that can produce original and meaningful outputs, such as text, images, audio, or video, based on patterns via algorithms from large existing datasets [1]. Large language models (LLMs) such as ChatGPT and Copilot demonstrate how GenAI functions as an advanced tool for information retrieval and problem-solving, supporting humans through intelligently formed, contextually aware responses [1].

There are both strong opportunities and challenges when considering GenAI’s potential to support patient care delivery, specifically in specialities managing complex chronic conditions [2,3]. Codesign with healthcare professionals and affected families is essential to enable the successful long-term implementation of these tools in clinical practice, maintain trust, and foster management of complex conditions. These advancements can be significant for paediatric rheumatology, a speciality where the need for patient and family engagement is crucial. Therefore, understanding healthcare professionals’ views of GenAI tools and their potential capabilities is vital in this clinician- and family-centred healthcare ecosystem, in which digital tools provide an additional opportunity to manage health conditions. Paediatric rheumatology encompasses a diverse range of chronic autoimmune and autoinflammatory diseases affecting multiple organs [4,5]. An estimated 6 to 7 million children have a rheumatological condition globally [6]. Juvenile idiopathic arthritis (JIA) is the most common rheumatological disease, with prevalence rates ranging from 3.8 to 400 of 100,000 [7]. High variability in prevalence rates is observed based on diagnostic and classification criteria, which account for differences in what is classed as a ‘case’ in addition to decisions around inclusion or exclusion of certain subtypes, study design, and sample size, as well as population and environmental factors, including healthcare access [7]. Paediatric rheumatological conditions are often lifelong and linked to functional limitations, with difficulty performing daily activities and overall decreased quality of life [8]. In addition, some paediatric rheumatological conditions are rare and can differ significantly between individual patients, making it challenging to predict the progression of the condition and effectively tailor treatment plans [9].

In adult rheumatology care, GenAI is already used to bring together medical information, interpret patient records, and support diagnostic reasoning and decision-making [[10], [11], [12]]. GenAI has also made advances in paediatric rheumatology, investigating avenues to support disease understanding, biomarker identification, imaging interpretation, and personalised treatment planning [13,14]. Although results seem promising and encouraging to date, showing good levels of diagnostic accuracy, with GenAI tools having the ability to process complex information and perform scientific reasoning with sensitivity, concerns relating to bias from dataset sources, and limited validation samples persist [13,14]. However, these developments demonstrate GenAI’s expanding role in undertaking increasingly complex, speciality-specific tasks; however, early applications of LLMs in healthcare are still primarily focused on information delivery, knowledge provision, and communication [15,16], rather than providing assistance with physical tasks.

Managing chronic paediatric rheumatological conditions extends beyond the healthcare setting [17], placing a significant burden on families, in particular, caregivers [18,19] who are often responsible for everyday disease management like administering injections [20], supporting exercise regimens [21], and dealing with the unpredictable nature of recurring flares [22]. In addition, limited access to paediatric rheumatology specialists poses another significant challenge [[23], [24], [25]]. Ongoing workforce shortages have led to delayed access to multidisciplinary care alongside added pressure on families to manage treatments at home [[23], [24], [25]]. Moreover, managing childhood rheumatological conditions causes burden and disruption to everyday life because families often travel long distances to their specialist appointments, which impacts schooling, caregiver work, and has financial implications [26]. Beyond physical symptoms, living with rheumatological conditions is associated with impaired psychosocial well-being [[27], [28], [29]]. These challenges emphasise the need for supportive solutions, potentially including GenAI tools, which can extend beyond clinical appointments, strengthen continuity of care, alleviate the burden on families and patients, and provide additional support to healthcare professionals in paediatric rheumatology.

The use of GenAI may reshape healthcare professionals’ (HCPs’) relationships with patients, raising both ethical and practical challenges [30]. HCPs’ perceptions and attitudes towards GenAI are critical in determining its adoption as their willingness to integrate such systems directly shapes clinical uptake, patient trust, and the safe, effective use of GenAI in practice [[30], [31], [32]]. Therefore, workforce education is required when considering GenAI adoption in clinical care. Successful GenAI adoption in healthcare depends not only on the capabilities of the technology but also on how it is understood, trusted, and embedded within clinical workflows. Studies show that HCPs’ perceptions and behaviours are influenced by factors such as perceived benefits, trust and transparency, and the impact on the clinician-patient relationship [[32], [33], [34], [35], [36]]. In addition, there are ethical concerns about data privacy, liability, accuracy, misdiagnosis, and depersonalisation, emphasising that GenAI’s integration must be cautious and context-aware [33,37]. There is also apprehension that reliance on GenAI may erode clinical skills, foster dependency on GenAI outputs, and diminish professional autonomy [34,37,38].

In a recent systematic review, 19 studies identified GenAI’s perceived lack of empathy and human touch as a limiting factor [38]. In home care contexts, the stakes of GenAI use are heightened. For example, if a chatbot incorrectly guides a parent during a joint exercise or offers false reassurance about inflammation, the result could be delayed intervention or an exacerbated flare. Such scenarios raise unresolved questions of liability and supervision. Issues of privacy, data security, and even emotional dependency, such as families placing more trust in chatbots than in clinicians, also remain underexplored; thus, developing GenAI-specific information governance guidelines is critical. Ethical and legal challenges include maintaining data privacy, ensuring informed consent, defining accountability for GenAI-driven judgements, and mitigating bias in outputs [[37], [38], [39], [40], [41], [42]]. The need for policies around safety, accountability, and transparency is clear. Moreover, research suggests that experienced nurses outperform advanced GenAI systems in areas requiring high-level clinical reasoning rooted in professional experience and hands-on care, suggesting that GenAI still needs human oversight in clinical care [43].

Successful adoption of GenAI tools depends heavily on HCPs’ acceptance and level of trust, given their role in ensuring patient safety and maintaining quality of care [44,45]. Not fully understanding HCPs’ concerns and expectations of GenAI tools means that these tools may be designed and implemented in ways that misalign with realistic needs and values, ultimately limiting effectiveness and sustainability, especially in relation to complex long-term conditions [46].

Considering both the potential and challenges, there remains a clear gap in the literature exploring how HCPs perceive the use of GenAI to support physical, hands-on care tasks at home in paediatric rheumatology, where such tasks are both emotionally and technically complex. As an initial exploratory study, the focus was intentionally limited to HCPs’ perspectives to better understand perceived clinical utility, feasibility, and implementation considerations before exploring patient and family perspectives in future work.

## METHODS

### Study population

HCPs working in paediatric rheumatology were invited to join a focus group as part of the IMPACT study (Interventions to improve Mental health support in families with children and young people with Chronic rheumaTological conditions) [47]. The IMPACT study aimed to design, develop, and test a chatbot intervention for caregivers of children and young people with paediatric rheumatological conditions to provide psychosocial support for families [47]. The IMPACT study used Experience-Based Co-Design throughout all phases of the study, enabling a large Steering group of children and young people (*N =* 4), parents and carers (*N =* 4), as well as HCPs (*N =* 3) to be involved in Patient and Public Involvement and Engagement (PPIE) and research activities, as well as decision-making processes. For more information about our PPIE involvement, please visit our website (https://www.theimpactstudy.co.uk/). Although the original pilot study developed a chatbot relying on limited GenAI before GenAI was readily available and accessible—particularly within the healthcare remit—the goal was to understand how support, functionality, and content could be increased by adding AI into the chatbot in the future. Therefore, the current project wanted to identify HCPs’ views on using GenAI to support families with hands-on care. Paediatric rheumatology HCPs were recruited from 14 hospital trusts from the wider multidisciplinary team (MDT) with the aim of including a range of professions such as consultants, nurses, physiotherapists, psychologists, and others. Ethical approval was granted by the National Health Service (NHS), Health Research Authority (HRA), and Health and Care Research Wales (HCRW; REC reference: 23/YH/0172). Full informed consent was obtained from all participants, including consent for sharing anonymised quotes. The recruitment strategy included email invitations that were sent out to external centres participating in the IMPACT study, word of mouth, and an advert on our study website (https://www.theimpactstudy.co.uk/).

### Study design and data collection

This project used a qualitative design to capture HCPs’ views on families using GenAI to support at home. Participants were invited to join 1 of 7 focus groups. Consolidated Criteria for Reporting Qualitative Studies [48], a comprehensive 32-item checklist, was used to report on and evaluate the study design (Supplementary Material S1 to review the checklist).

The project used a vignette-based methodology to help facilitate conversations and engagement following the 3 principles outlined in a scoping review by Tremblay et al [49]. These 3 principles improve rigour by: (1) defining the methodology of using a vignette-based approach, (2) providing information on the development, and (3) specifying how it is used to collect qualitative data.

### Defining the methodology

Vignette-based methodology encompasses a range of modalities and can be used to present clinical case reports or case studies, hypothetical scenarios, or training materials [49]. Moreover, vignettes are commonly used to elicit reflections on hypothetical accounts from an expert audience [49]. In this study, the methodology was used to elicit data on perceived views relating to 3 hypothetical scenarios of how GenAI could benefit families with hands-on care management. Drawing on personal views in relation to work experience, alongside expertise in patient care, as well as personal opinions relating to GenAI, was crucial to address the research question.

### Development

The vignette development included involvement of expert HCPs who consulted on the scenarios that were chosen (Supplementary Material S2 for a full version of the vignettes and introductory slides that were used). The facilitator, SS, who was an independent researcher and not affiliated with the hospital trust, devised a set of open questions that were asked to elicit an open discussion to obtain feedback and responses. SS was chosen to be the facilitator to reduce bias as she had no pre-existing relationship with the participants. SS was involved in this project as part of her master’s programme at University College London, which she disclosed in the introduction section of each focus group. Open questions included (1) ‘How realistic or helpful does this seem based on your clinical experience?’, (2) ‘What kinds of concerns might this raise and what advantages do you see?’, and (3) ‘Would this kind of AI support help or hinder the caregiver–patient relationship?’ Prompts to further explore benefits and challenges were used where needed. The structure of questions was flexible, and prompts were used to promote and maintain the flow of conversation if necessary.

Purposive sampling was used to recruit HCPs from different backgrounds to get views and expert opinions from members of the wider MDT. They were invited to attend 1 of 7 virtual focus groups, which were held on Microsoft Teams and presented with 3 vignettes. These were introduced to participants for the first time during the focus group, with participants having not seen these before. The focus groups were recorded via Teams, and transcripts were downloaded securely to aid data analysis.

The 3 scenarios that were included were:

(1) A family using a GenAI tool to assist with weekly methotrexate injections at home. They may feel uncertain, even if they have been shown how to do it.
(2) A parent who is concerned that their child may be experiencing a flare-up. They use a GenAI assistant to guide them through a symptom checker at home.
(3) A child with JIA needs support with daily physiotherapy exercises. At home, the family uses a GenAI aide to guide their child through their routine exercises.

### Data collection

Written informed consent was obtained from all participants before receiving a Teams link for their chosen focus group session. After a brief introduction from the facilitator (SS), each scenario was presented using Microsoft PowerPoint for approximately 10 minutes and was visible throughout the discussion. Each slide presented a few bullet points exploring possible ways in which GenAI could support hands-on care. For example, for scenario 1, ideas of how methotrexate injections can be facilitated were provided by giving advice on positioning and technique accuracy with the help of a mobile phone camera.

The facilitator (SS) remained neutral and open and ensured that all participants had an opportunity to speak. For consistency, the facilitator remained the same for all focus groups. Sociodemographic data, such as age, gender, profession, and length of service in practice (overall as well as in paediatric rheumatology), were requested via a brief online survey link. The facilitator (SS) was supported by the Chief Investigator (PL) and IMPACT study Research Facilitator (KK) during the focus group sessions; a member of the IMPACT Steering group was also present.

### Data analysis

To analyse qualitative data, transcripts were pseudoanonymised. As direct quotes are used to present data, which are classed as personal data, pseudoanonymisation allows data to be shown safely in line with the General Data Protection Regulation by removing identifiable information. All transcripts were analysed qualitatively using NVivo 14.23.3 (Lumivero). Inductive thematic analysis, as originally proposed by Braun and Clarke in 2006 [50], was used to code the data and identify the themes. Grounded theory-guided analysis, as an inductive approach, was chosen to explore new frameworks, as the themes that were analysed were strongly linked to the data itself, rather than using pre-existing theoretical frameworks or preconceptions. The 6 principles of Braun and Clarke’s approach to thematic analysis were followed: (1) data familiarisation, (2) generating initial codes, (3) searching for themes, (4) reviewing themes, (5) defining and naming themes, and (6) producing the report. As qualitative analysis is an active process, themes were shaped over time. Transcripts were independently coded by SS and KK. Conflict resolution involved a team meeting with all authors present. A majority vote was used to agree on codes. All themes were reviewed, refined, and agreed upon by both KK and PL. A range of exemplar quotes was selected, reviewed, and chosen by KK and PL unanimously. All researchers were female and received training in qualitative research, which was a key criterion for being allowed to analyse data for this study, with the Chief Investigator (PL) being an established qualitative researcher in investigating psychosocial burden experienced by children and families living with paediatric rheumatological conditions.

There are still no clear guidelines regarding how to determine sample and justify sample sizes in qualitative research. Most commonly, cut-offs to determine adequacy of purposeful sampling relate to saturation [51]. There is a more recent distinction to prioritise theoretical saturation over data saturation [51,52]. Theoretical saturation refers to the construct originally proposed in grounded theory that posits that where further data collection would not yield any new theoretical insights, data collection can be stopped [53]. The current study used an approach based on a predefined stopping criterion, which was set at 2, whereby when no new codes were identified in 2 consecutive focus groups after the predefined threshold of an initial sample of 4 focus groups, saturation was met, and data collection was stopped [52]. Descriptive analyses were carried out using the Statistical Package for Social Sciences to analyse demographic data.

## RESULTS

Data were collected between June and August 2025, and participants were invited to join 1 of 7 focus groups. As saturation was reached using the stopping criterion outlined above, with no new codes identified after focus group number 5 (groups 6 and 7 did not yield any new codes), recruitment was closed, and data collection was stopped. This led to a sample of 37 HCPs (83.8% female) who participated in the study with an average of 11 years in paediatric rheumatology (SD = 9.19). A full set of demographics can be found in Table 1. There were 4 to 9 participants in each group with an average time of 27 minutes per session (ranging between 22 and 31 minutes).

**Table 1:** Demographics of participants

Variable	M (SD)	Range (in years)	
		Min	Max
Age (*N* = 32)	44.25 (11.59)	25.0	65.0
Years in Practice (Overallpractice [overall]) (*N* = 37)	20.75 (11.87)	4.4	45.0
Years in Practicepractice (in Paediatric Rheumatologypaediatric rheumatology) (*N* = 37)	11.23 (9.19)	0.0	36.0
Variable (*N* = 37)	Percentages		
Gender			
Gender			
Female	83.8%		
Male	16.2%		
Ethnicity			
White	89.2%		
Any other ethnic group	10.8%		
Job roles			
Consultants (paediatric rheumatologists)	18.9%		
Resident doctors/SHOs*	8.1%		
CNS**/ANP***/ward nurses	45.9%		
Physiotherapist/PT**** assistants	13.6%		
Clinical Psychologists & Occupational Therapists	8.1%		
Research staff	5.4%		
Clinical psychologists and occupational therapists	8.1		
Research staff	5.4		

ANP, advanced nurse practitioner; CNS, clinical nurse specialist; PT, physiotherapy; SHO, senior house officer.

### Themes

Six main themes were identified: (1) opportunities of GenAI to benefit patient care; (2) disadvantages and challenges; (3) risks, safety netting and evaluation; (4) different approaches of utilising GenAI; (5) role of HCPs in the world of GenAI; and (6) ensuring inclusivity and accessibility. A full list of themes and subthemes supported by exemplar quotes can be found in Table 2.

**Table 2:** A full list of themes and subthemes alongside exemplar quotes

Main theme		
*Subthemes*	Quote #	Quotes
Theme 1: opportunities of AI to benefit patient care		
*Giving more guidance*	Q1	‘I mean this would be a physio’s dream. At the moment, they’re given a little sheet, you know, obviously, they go through it with the physio and then they’re expected to go away and remember this. So, to have the sort of visual representation and, also, the reminders and to make sure that they’re actually doing it correctly, I think it’s just going to make everyone’s life easier’. HCP 13, age unknown, Clinical Nurse Specialist
	Q2	‘I do find that parents, they kind of worry, if they’re doing the exercises correctly at home. You know, they leave a busy clinic, they’ve been given a lot of information about blood results, medication, lots of stuff, and then they come home and say, OK, what was the exercise? Are we doing it right? They’re worried that if they overdo it or under do it, maybe they’re going to make their child’s arthritis worse’ HCP 11, aged 50-59, Advanced Nurse Practitioner
*Providing reassurance*	Q3	‘When you’re first given your drug education, it’s such a lot of information to take in, trying to remember all that stuff and feel like you remember it… but when you actually go to do the injection, I would definitely, if it was me having to do this, want that reassurance to be able to sort of check back and not feel like I’ve forgotten everything that the nurse or doctor has told me’. HCP 26, aged 30-39, Research Coordinator
	Q4	‘I think families would be reassured if they knew the technique that their child was doing was correct when they are doing the home exercise programme’. HCP 6, age unknown, Consultant
	Q5	‘A lot of families, once they’ve had their appointment, whether it’s a new diagnosis or new medication, they often come away feeling overwhelmed, and sometimes when you ask them, they don’t remember or recall details about their medication or exercise, so it could be helpful in that sense’. HCP 2, aged 20-29, Physiotherapy Assistant
*Motivational and engaging*	Q6	‘For Physio, things could be gamified so that you’re collecting points or collecting tokens or working towards different things with your compliance… I’m sure a lot of problems with compliance with these types of exercises is that they aren’t particularly fun. You don’t get necessarily that kind of endorphin kind of buzz that other types of sport or activities might give you. So, being able to kind of gamify it, and make it really, really kind of fun collecting stuff, you’ll have a degree of patients that are going to enjoy seeing their own data. They’ll get a buzz and the kick out of seeing how they’ve progressed, you know, so it’s going to be really useful’. HCP 37, aged 40-49, Senior Physiotherapist
	Q7	‘Encouragement and reminders…that would be helpful’. HCP 3, aged 50-59, Consultant
*Future opportunities/potential*	Q8	‘I was wondering also if they couldn’t get something right, would there be an option to give an alternative exercise that targets the same area but may be easier for that child to do’. HCP 6, age unknown, Consultant
	Q9	‘We find a lot of the time that they may say yes, I do my exercises, but they kind of rush through all the repetitions. They don’t do them effectively, so, a tool that can count slowly for them, so they can keep in time with the counting would be helpful’. HCP 2, aged 20-29, Physiotherapy Assistant
	Q10	‘Can I ask if it’s possible that we could also [have a tool for] blood monitoring for kids who need their bloods done, … or maybe diet [as well]’. HCP 11, aged 50-59, Advanced Nurse Practitioner
Theme 2: disadvantages and challenges		
*Scope of AI*	Q11	‘I guess what worries me is how precise it actually is. We know that there’s so many different compensatory movements that it may not pick up we could with hands-on [assessments]’. HCP 2, aged 20-29, Physiotherapy Assistant
	Q12	‘We don’t usually recommend people logging pain because it makes them focused on it. So, I don’t know, that’s just something that we tend not to recommend for kids’. HCP 34, aged 40-49, Clinical Psychologist
	Q13	‘Would you tell them what to do then? After [the joint assessment], does it offer a summary and guidance. So would [the AI aide] say yes, this is a flare. You should contact your local clinician, or would it go as far as saying you should take some naproxen or ibuprofen?’ HCP 6, age unknown, Consultant
*Variation in care*	Q14	‘With anything clinical we always go by guidelines specific to the hospital and sometimes, there can be variation of how you give an injection, even between 2 different nurses, let alone a different country, or speciality’ HCP 24, aged 20-29, Ward Nurse
*Pressures of AI—‘Big Brother is watching’*	Q15	‘And also, it could put them off doing [their exercises] if they need motivation and encouragement, really. It depends on the AI, how they respond if they’re doing the wrong technique consistently. When they’re watching the video and they can’t get it right, will that make them not want to do it at all and avoid doing it?’ HCP 1, aged 40-49, Occupational Therapist
	Q16	‘I agree with that frustration because I even think of when phones ask me to do something and I can’t get it right; it really frustrates me. So, I can imagine if it’s difficult to get it right that they may not do it’. HCP 14, 40-49, Senior Physiotherapist
	Q17	‘Parents might be put off knowing the camera is monitoring their movement’. HCP 1, aged 40-49, Occupational Therapist
*Overreliance*	Q18 Q19	‘I suppose my biggest concern when I look at something like this is I can see parents that would make their child do [a joint assessment] on a daily basis so it may be a little bit over the top and then I would also be worried that although they may think they’re doing an assessment properly, it’s not going to replace of a health professional….and then we’re missing or overdiagnosing something that actually isn’t there’. HCP 14, aged 40-49, Senior Physiotherapist
	Q19	‘My concern is there’s a lot of parental anxiety, a lot of the time. If you give them a tool like this, they’re just going to be constantly on the tool trying to figure out what’s going on. And then then then say into, as you know, well, I’ve used the tool and it says it’s definitely a flare of arthritis. I need to get seen … It might just be that some families just might get a bit consumed and obsessed by it, and that’s the only risk that I can potentially see’. HCP 4, aged 40-49, Clinical Nurse Specialist
*Procedural anxiety*	Q20	‘I think with some of the younger patients, they just want to get the injection kind of over with. So, I think by taking photos during the administration, they could get a bit more stressed and anxious waiting for the injection to be given’. HCP 9, aged 30-39, Clinical Nurse Specialist
	Q21	‘I do wonder if looking at a video and then step by step instructions and taking pictures almost draws the process out a little bit more for the child. I mean, I completely understand from a parent’s point of view, you know, they want to be getting it right and you don’t want to rush through [administering the injection] just to avoid anxiety. But I do wonder on the side of the child, whether some of this would increase kind of the focus on it and possibly make the process harder and more anxiety inducing for the child’. HCP 28, aged 40-49, Physiotherapist
*Boundaries*	Q22	‘I guess from an ethical point of view, I can see it from both sides. I agree not wanting to place burden and responsibility on parents like that… because I think when you have a sick child you’re many things to your child anyway, aren’t you but there’s a line between being a parent and a healthcare person, and I think sometimes it can get blurry enough with children that have chronic illness and sometimes actually they’re better off just being mum or dad. Equally, I can see that knowledge is power and knowing more can empower you and I think there’s a fine line to be crossed there. You know, I think there’s obviously some kind of ethical conundrums around accuracy and things like that, but I think it’s also about the role of caregivers and where that boundary lies, I don’t know the answer to it’. HCP 28, aged 40-49, Physiotherapist
Theme 3: Risks, safety netting and evaluation		
*Risks*	Q23	‘I think many of us who use generative models and large language models a lot understand that hallucination is still an issue despite all the best prompting techniques, but with some of the retrieval-augmented generation strategies, if you can properly curate the models so that they’re only applying tasks that have been approved, it should be OK, but that’s why I was saying you can’t, at this point, yet, we can just let GPT 5 run loose with this. Who knows what it would do? You just wouldn’t want it to make mistakes, that’s all’. HCP 29, aged 60-69, Consultant
	Q24	‘I think there’s a lot of misconceptions about AI and certainly, I’ve had good experiences of using it but I’ve learned the hard way to be careful, and fact check stuff so’. HCP 28, aged 40-49, Physiotherapist
	Q25	‘The nature of some of these AI models is that it essentially learns the more times it’s asked something. So, you can actually get a different response or slightly different response depending on which time you’ve asked exactly the same question and I think that’s only going to drive anxiety, which is one of the issues mentioned and it may cause confusion and therefore error’. HCP 3, 30-39, Consultant
*Ethical considerations and data protection*	Q26	‘The camera is tracking their movements, but what else might that camera do, and what else might it record and where does that information go, and who else has access to that information that has come from the camera?’ HCP 6, age unknown, Consultant
	Q27	‘How would the photos or videos that are taken be stored? I think privacy concerns would be a big issue, especially if it’s of children and of body parts’. HCP 26, aged 30-39, Research Coordinator
	Q28	‘Because they’ve consented to it, I don’t see an issue with it’. HCP 10, aged 40-49, Clinical Nurse Specialist
*Need for evaluation*	Q29	‘I think if this tool was validated and you know you could actually properly assess the joint angles and make sure that the physiotherapy at home is being done correctly, then I think this could be quite powerful. But it would, it would definitely need to be validated’. HCP 15, aged 30-39, Consultant
	Q30	‘I was just going to say it’d be amazing to measure the outcomes. You know, if this was up and running, I think it would make a huge difference for patients, a huge difference for physios who are so pushed for time and a huge difference to the NHS as well financially because patients wouldn’t need to be seen as much. So yeah, I can’t see it being anything positive’. HCP 13, age unknown, Clinical Nurse Specialist
	Q31	‘I would like to see a bit of a comparison, because it would be interesting to see the comparison between the performance of the human, and AI, because so far the assessments of joint counts by AI are really, really encouraging and really surprising but this is in rheumatoid arthritis’. HCP 27, aged 20-29, Research Fellow
Theme 4: Different approaches of utilising AI		
*AI as educational tool*	Q32	‘It certainly could be useful to have the education accessible to them’. HCP 22, aged 30-39, Clinical Psychologist
	Q33	‘I see patients in clinics and patients do comment on the difficulty of giving injections, so it could be useful for health professionals to signpost to this’. HCP1, aged 40-49, Occupational Therapist
*To complement care*	Q34	‘A quick reference tool for families at home. I think it would be really good because I definitely agree families are likely to forget or have a panic and just completely mind blank on how to give an injection. So, for them to have something to go to, it would be good as a reference side; I wouldn’t necessarily use it as a standalone. You would always want some sort of nursing input rather than just going straight to the AI tool’. HCP 4, aged 40-49, Clinical Nurse Specialist
	Q35	‘It would be useful to have education accessible to them, and it could be framed as, while you await your clinical assessment here are some reminders and tips of what you can do to self-manage at home’. HCP 22, aged 30-39, Clinical Psychologist
	Q36	‘It could help with the queries we get, avoid a frantic phone call or email if they go straight to this for general information’. HCP 4, aged 40-49, Clinical Nurse Specialist
*Two-way communication tool with HCPs*	Q37	‘It could be quite a useful screening tool if they’re sending pictures and videos for the clinicians to decide whether it’s relevant for them to be booked in, like an emergency clinic, I guess, or whether they can wait to their next appointment perhaps’. HCP1, aged 40-49, Occupational Therapist
	Q38	‘I also like the idea of spying and tracking adherence because nobody does [exercises]. I’m a physio, and I have my own physio prescribed, and I don’t do my exercises’. HCP 28, aged 40-49, Physiotherapist
Theme 5: Role of HCPs in the world of AI		
*AI as perceived threat*	Q39 Q40 Q41	‘I’m not concerned about our roles just yet’. HCP 11, aged 50-59, Advanced Nurse Practitioner ‘You can’t replace nurses. People need people’. HCP 16, aged 50-59, Clinical Nurse Specialist ‘I do worry that we will lose our role to AI eventually. Hopefully I’ll be nearing retirement by the time it happens’ HCP 4, aged 40-49, Clinical Nurse Specialist
*Human expertise and experience*	Q42	‘The subtle changes…, sometimes it’s tricky for families at home to assess that and I think nothing can equate to coming in and having that hands-on review as well’. HCP 17, aged 40-49, Clinical Nurse Specialist
	Q43	‘In young people with arthritis it’s sometimes a subtle restriction in the joints that you see on examination that actually indicates active disease, so you can’t substitute the clinical assessment’. HCP 15, aged 30-39, Consultant
	Q44	‘I think for me, I think it’s very hard for parents to palpate joints in the way that health professionals do because it does take quite a bit of training to actually do it and I suppose I’d be a bit worried about this. I’d like to see some data on whether it actually produces the right results or not, if it’s false negatives, false positives and what that does to the patient journey, if there is a flare going on and whether it delays them, if it’s a false negative’. HCP 32, aged 60-69, Consultant
	Q45	‘It makes an assumption that parents are very objective in terms of the description of what’s going on for their child but there’s so many other factors in terms of their worry and their own background and their journey up to that point and some parents really kind of magnify their concerns whereas others kind of minimise it, so it might be difficult for a computer to act on it’. HCP 34, aged 40-49, Clinical Psychologist
*Workload support*	Q46	‘If used in the right way, I believe it could be a good supporting system. However, I don’t think it would ever actually replace the human interaction’. HCP 30, aged 20-29, Senior House Officer
	Q47	‘Well, I suppose from our point of view with the, with the nurses having already a high workload, this would take away some of the workload that they’re dealing with in a in a daily basis where and then they can concentrate more on patient care’. HCP 14, aged 40-49, Senior Physiotherapist
	Q48	‘We have home care nurses that might go in and do this kind of procedure for the first few times, walk a family through these kind of injections at home. And this would certainly save on resources and time if a model could be developed’. HCP 29, aged 60-69, Consultant
Theme 6: Ensuring individuality and accessibility		
*Health literacy*	Q49	‘It might also be helpful for people where English isn’t their first language, because it’ll be a very visual tool’. HCP 18, aged 60-69, Lead Nurse
	Q50	‘It’ll only meet the needs of certain family groups as lots of families don’t have the health and literacy tools to navigate AI’. HCP 16, aged 50-59, Clinical Nurse Specialist
	Q51	‘There are lots of language barriers at our hospital, so if this was part of AI it would be useful for families to have translations that are accurate and accessible’. HCP 24, aged 20-27, Ward Nurse
*Accessibility*	Q52 Q53	‘I come across some families, and they travel an hour, or 2 hours, just to get the physio piece done, because they feel that comfort or that reassurance that they’re doing it correctly. So yeah, this would be good’. HCP 11, aged 50-59, Advanced Nurse Practitioner
	Q53	‘I think the benefits are that’s it’s easily available, at a time that a parent wants it, whereas you’d have to wait till the CNS (Clinical Nurse Specialist) is available’. HCP 32, aged 60-69, Consultant
*Tech literacy*	Q54	‘I’m not sure that people would understand the process, and it might actually create anxiety, because what I’ve noticed in my practice is that people are a lot less tech oriented, than you think. You need to be careful not to overestimate people’s skills in using AI. That’s something that I think is really important’. HCP 3, aged 50-59, Consultant
*Individuality and inclusivity*	Q55	‘AI is basically very manufactured. It does not allow for individuality whereas I certainly taper my nursing to that individual family and their needs’. HCP 4, aged 40-49, Clinical Nurse Specialist
	Q56	‘I think also making sure that it works on people with different skin colours is important, because that’s always, historically been a big issue that people who aren’t white basically aren’t represented in medical textbooks. So, with the AI, it should have enough sort of education on different people’. HCP 26, aged 30-39, Research Coordinator
*Choice*	Q57	‘I think if the family is comfortable with using this this method, then that’s fine for me because it’s like I said, different things work for different families better’. HCP 7, age unknown, Clinical Nurse Specialist

AI, artificial intelligence; CNS, Clinical Nurse Specialist; HCP, healthcare professional; NHS, National Health Service; Q, quote; RAG, retrieval-augmented generation.

#### Theme 1: opportunities of AI to benefit patient care

HCPs identified a range of opportunities in which GenAI could benefit patient care. HCPs agreed that receiving and having to manage a new diagnosis, including parental administration of injections, creates a stressful environment for some families, so additional support could provide a layer of reassurance and a means to sense-check their knowledge if they feel overwhelmed:

*‘A lot of families, once they’ve had their appointment, whether it’s a new diagnosis or new medication, they often come away feeling overwhelmed, and sometimes when you ask them, they don’t remember or recall details about their medication or exercise, so it could be helpful in that sense’. HCP 2, aged 30*-*39, Physiotherapist*

Moreover, HCPs liked the idea that these tools can facilitate support that can be accessed easily without causing any delays when having to wait for a health professional to respond or become available. They also discussed how these tools have the potential to reduce barriers and provide support, especially for families who live in more remote areas and may have to travel long distances to access care:

*‘I come across some families, and they travel an hour, or 2 hours, just to get the physio piece done, because they feel that comfort or that reassurance that they’re doing it correctly. So yeah, this would be good’. HCP 11, aged 50*-*59, Advanced Nurse Practitioner*

Some HCPs also believed that using such tools could improve compliance by being motivational and engaging, and provided future scenarios for support, such as blood monitoring and diet.

#### Theme 2: disadvantages and challenges

HCPs questioned the scope of what GenAI could deliver by considering where the boundaries should lie. HCPs contemplated the role of GenAI tools and whether they should have a more passive role as an informational support tool providing guidance, or whether they could have the capacity to provide alternative exercises or even advice on treatment options and next steps:

*‘After [the joint assessment], does it offer a summary and guidance. So would [the AI aide] say yes, this is a flare. You should contact your local clinician, or would it go as far as saying you should take some naproxen or ibuprofen?’ HCP 6, age unknown, Consultant*

As the scenarios were hypothetical, HCPs asked questions relating to potential precision, depth, flexibility, and accuracy. In addition, some HCPs thought that families might feel pressured due to constant monitoring from GenAI, frustration if they were unable to follow instructions, or if their child fails to follow an exercise technique correctly, which may put them off doing their exercises. Some were worried that families may become consumed by these tools, over-relying on them:

*‘My concern is there’s a lot of parental anxiety, a lot of the time. If you give them a tool like this, they’re just going to be constantly on the tool trying to figure out what’s going on. And then say into, as you know, well, I’ve used the tool and it says it’s definitely a flare of arthritis. I need to get seen’. HCP 4, aged 40*-*49, Clinical Nurse Specialist*

HCPs were also concerned that relying on GenAI may draw out processes such as home administration of medication injections that are already anxiety-inducing for many children and young people and may cause additional harm to their well-being:

*‘I do wonder if looking at a video and then step by step instructions and taking pictures almost draws the process out a little bit more for the child. I mean, I completely understand from a parent’s point of view, you know, they want to be getting it right and you don’t want to rush through [administering the injection] just to avoid anxiety. But I do wonder on the side of the child, whether some of this would increase kind of the focus on it and possibly make the process harder and more anxiety inducing for the child’. HCP 28, aged 40*-*49, Physiotherapist*

HCPs also discussed the role of caregivers and thought that asking caregivers to conduct joint examinations could blur the boundaries between the roles of healthcare staff and caregivers who are already heavily involved in looking after children with complex medical needs.

#### Theme 3: risks, safety netting and evaluation

HCPs acknowledged that, in order for GenAI tools to provide adequate support to families, risk assessments should be conducted to ensure that the technology is safe and appropriate. HCPs shared their worries relating to accuracy, specifically in relation to hallucinations, a concept whereby GenAI is able to fabricate information or return wrong information from the internet [2]. Privacy, data protection, and storage concerns have also been identified:

*‘The camera is tracking their movements, but what else might that camera do, and what else might it record and where does that information go, and who else has access to that information that has come from the camera?’ HCP 6, age unknown, Consultant*

HCPs highlighted the need for evaluation of these tools to maximise their potential:

*‘I was just going to say it’d be amazing to measure the outcomes. You know, if this was up and running, I think it would make a huge difference for patients, a huge difference for physios who are so pushed for time and a huge difference to the NHS (National Health Service) as well financially because patients wouldn’t need to be seen as much’. HCP 13, age unknown, Clinical Nurse Specialist*

#### Theme 4: different approaches of utilising AI

HCPs considered different ways in which GenAI tools could be used. Opinions varied, including whether such tools should be used as stand-alone tools providing informational content, as an adjunct to complement clinical care, or as a 2-way communication aid between families and health professionals.

*‘It would be useful to have education accessible to them, and it could be framed as, while you await your clinical assessment here are some reminders and tips of what you can do to self-manage at home’. HCP 22, aged 30*-*39, Clinical Psychologist*

*‘It could help with the queries we get, avoid a frantic phone call or email if they go straight to this for general information’. HCP 4, aged 40*-*49, Clinical Nurse Specialist*

Although some HCPs thought it would be a good idea to have these tools available as reference tools, others thought that they could provide a means to monitor compliance.

*‘I also like the idea of spying and tracking adherence because nobody does [exercises]. I’m a physio, and I have my own physio prescribed, and I don’t do my exercises’. HCP 28, aged 40*-*49, Physiotherapist*

The idea of GenAI being utilised as a screening tool was also considered:

*‘It could be quite a useful screening tool if they’re sending pictures and videos for the clinicians to decide whether it’s relevant for them to be booked in, like an emergency clinic, I guess, or whether they can wait to their next appointment perhaps’. HCP1, aged 40*-*49, Occupational Therapist*

#### Theme 5: role of HCPs in the world of GenAI

GenAI was discussed in the context of being a threat to job roles; however, most HCPs did not think that this is an issue just yet:

*‘I do worry that we will lose our role to AI eventually. Hopefully I’ll be nearing retirement by the time it happens’ HCP 4, aged 40*-*49, Clinical Nurse Specialist*

*‘In young people with arthritis it’s sometimes a subtle restriction in the joints that you see on examination that actually indicates active disease, so you can’t substitute the clinical assessment’. HCP 15, aged 30*-*39, Consultant*

Moreover, the majority of HCPs seemed content to work alongside GenAI as a support tool, but cautioned that it would never replace human expertise and face-to-face assessments:

*‘If used in the right way, I believe it could be a good supporting system. However, I don’t think it would ever actually replace the human interaction’. HCP 30, aged 20*-*29, Senior House Officer*

#### Theme 6: ensuring inclusivity and accessibility

HCPs are advised to be cautious about overestimating families’ tech and health literacy skills, but thought it may be a helpful tool to overcome language barriers:

*‘It’ll only meet the needs of certain family groups as lots of families don’t have the health and literacy tools to navigate AI’. HCP 16, aged 50*-*59, Clinical Nurse Specialist*

*‘There are lots of language barriers at our hospital, so if this was part of AI it would be useful for families to have translations that are accurate and accessible’. HCP 24, aged 20*-*27, Ward Nurse*

They also identified a risk to individuality and person-centred care, while also having the potential to exacerbate disparities when underrepresented groups are insufficiently represented in training data:

*‘AI is basically very manufactured. It does not allow for individuality whereas I certainly taper my nursing to that individual family and their needs’. HCP 4, aged 40*-*49, Clinical Nurse Specialist*

*‘AI needs to also make sure their information works for people with different skin colours, because historically this has been a big issue that people who aren’t white aren’t represented in textbooks and they may miss information on rashes on other symptoms’. HCP 26, aged 30*-*39, Research Coordinator*

However, ultimately, they agreed that families should be given the choice about whether they would want to use GenAI-enabled tools for additional support and appreciated the fast-changing technology landscape.

*‘I think if the family is comfortable with using this method, then that’s fine with me, because different things work for different families’. HCP 7, age unknown, Clinical Nurse Specialist*

## DISCUSSION

The aim of the current study was to understand HCPs’ views on families using GenAI to support hands-on care at home. Moreover, HCPs’ perceptions of hypothetical scenarios of what GenAI may be able to offer in the future were investigated, rather than a true reflection of what is currently available, addressing the potential scope of GenAI’s rapidly progressing and accelerating capabilities. Overall, the study found that HCPs welcomed the idea of families using GenAI tools to receive additional support between appointments; however, they expressed uncertainty around feasibility, accuracy, and GenAI’s capability and scope.

Different views of acceptability were observed depending on which of the 3 set scenarios HCPs were asked to consider caregivers: (1) administering subcutaneous injections (quotes Q3, Q5, Q20, Q21, Q33, Q34), (2) assessing joints for flares (quotes Q13, Q18, Q19, Q42-Q44), and (3) complying with exercise regimes (quotes Q1, Q2, Q4-Q9, Q38). HCPs favoured the third scenario and thought that families would benefit from additional support with their physiotherapy exercises. They thought that it could benefit families by being motivational, and children and young people may like the tool if it uses gamification (Q6), making exercises more engaging, which could in turn increase compliance (Q7). Lack of enjoyment and motivation to do exercises as they are perceived as boring and low priority has been identified as a barrier to adherence when interviewing children and young people [19]. Mixed views were observed for the first scenario. Although HCPs agreed that additional support may be suitable for some parents who administer injections by providing a layer of reassurance, allowing them to fact check and refamiliarise themselves with the correct procedure (Q33, Q34), they cautioned that this support may only benefit the parent, rather than the child themself (Q21). Children and young people with rheumatological conditions often suffer from needle anxiety, and HCPs felt that using the tool to assess a suitable injection site would draw out the procedure unnecessarily for the child and increase their distress (Q20, Q21). HCPs did not favour the second scenario and thought it blurred the lines between being a caregiver and a clinician (Q22). Concerns were raised as parents and carers do not receive adequate training to conduct joint assessments and may not be able to pick up on nuances that they would as health professionals, putting children at risk of caregivers misdiagnosing, overdiagnosing, or underdiagnosing flares (Q18, Q19).

Although HCPs liked the idea of providing additional support and guidance to families to support them with care between appointments, concerns were raised relating to accuracy, data protection and storage, privacy, and consent. They questioned the scope of these tools and expressed uncertainty around guardrails and safety netting, and agreed that they would benefit from evaluation and codesign to ensure they deliver to a standard that prioritises patient safety. A systematic review assessing management of chronic conditions in adults with the help of LLMs and GenAI found that accuracy and reliability pose ongoing challenges that need to be addressed, with misinformation in the form of hallucinations remaining a continued risk [2,54]. Moreover, readability and level of understanding were highlighted as a concern in current GenAI tools [54]. However, GenAI tools have the potential to reduce health inequalities, bridging the health literacy gap by providing health education, access to information for varying degrees of reading abilities, and even acting as a translator [55]. To address these challenges, Poalelungi et al [3] highlighted the need for a collaboration of technology developers alongside HCPs, researchers, and policy makers to enable the successful implementation of GenAI tools in a responsible, ethical, safe, equal, and inclusive manner. Understanding barriers to successful adoption is essential to developing suitable, effective, safe, and inclusive interventions and support tools for children and young people (CYP) and their families living with rheumatological conditions. This is crucial as these tools have the potential to reduce caregiver burden and uncertainty and improve the quality of life of families managing chronic conditions. In addition, paediatric rheumatology has a high demand for out-of-clinic communication with healthcare teams. Families frequently seek general information and advice, answers to medication queries, help with school, and transition problems, as well as to request psychosocial support [17]. Moreover, workforce pressures and shortages are commonly observed [[23], [24], [25]]. Therefore, developing tools to support families between appointments is needed, and GenAI interventions may provide a promising solution to aid families in managing their child’s care and, in turn, reduce pressures on the healthcare system.

HCPs disagreed over whether such tools should be available as stand-alone tools to provide information, education, and guidance, or whether these tools should allow 2-way communication between HCPs and families to help with the management of clinical care and, for example, facilitate communication relating to flares and booking of *ad hoc* emergency appointments where needed. The findings from the present study have implications for research and clinical practice. Understanding users’ needs and HCPs’ attitudes towards GenAI to facilitate care is important to inform clinical care and reduce harm [33,37]. Therefore, using codesign methodologies when implementing GenAI in clinical care is crucial. At present, GenAI tools require further development and testing before they are deemed safe to be rolled out into the clinical service. Ethical considerations and safeguarding of patients need to be prioritised, and risk monitoring and management need to be addressed when developing policies to allow successful implementation of GenAI in healthcare. Future research should also include CYP with rheumatological conditions alongside parents and carers to understand their needs in managing chronic conditions with the help of digital tools and identify barriers to and facilitators of GenAI tool utilisation in healthcare.

### Strengths

HCPs from 14 hospital trusts were included in the study, and the professionals involved all care for families from diverse backgrounds, including different cultural and socioeconomic backgrounds, living in different geographical locations. This strengthens the confidence in the transferability of our findings.

### Limitations

Although using purposive sampling allowed a range of professionals from the wider MDT to participate and focused on including a wide spectrum of experience, ranging from novice clinicians to those with a wide breadth of knowledge and experience, this led to some characteristics, such as gender and ethnicity, being neglected, and, therefore, some opinions may be underrepresented in this sample. This also includes an imbalance of professions, as we had a predominantly nursing-led sample (almost 46%). It is important to note that all 3 scenarios that were discussed and considered in the current study were all hypothetical. GenAI tools supporting hands-on care to manage paediatric chronic conditions do not readily exist and have not been approved by relevant health authorities to allow these to be used alongside clinical care. Another limitation is HCPs’ potential lack of knowledge relating to GenAI, which may have biased their opinions and could have led to increased caution and distrust in what GenAI can deliver.

## CONCLUSION

HCPs agreed that GenAI tools have the potential to provide additional support with hands-on care management for families living with paediatric rheumatological conditions, specifically to offer guidance and education, but also identified challenges to be addressed. GenAI tools in their current state are fallible with concerns around accuracy, reliability, inclusivity, privacy, data protection, and storage. Overcoming these challenges is crucial to being able to provide support to families and reduce the illness burden. Moreover, understanding HCPs’ views when developing standardised guidelines to support GenAI use is essential to advance their successful implementation.

## CRediT authorship contribution statement

**Klaudia H. Kupiec:** Writing – review & editing, Writing – original draft, Project administration, Methodology, Formal analysis, Data curation. **Sabina Sweet:** Writing – review & editing, Formal analysis, Data curation. **Sandrine Compeyrot-Lacassagne:** Writing – review & editing, Formal analysis, Data curation. **Polly Livermore:** Writing – review & editing, Validation, Supervision, Methodology, Investigation, Funding acquisition, Formal analysis, Data curation, Conceptualization.

## Competing interests

All authors meet the journal’s criteria for authorship, and none of the authors has any conflict of interest to declare. PL is currently receiving a personal fellowship award from the National Institute for Health and Care Research (NIHR) fellowship for nonmedical healthcare professionals (ACAF reference number 302864), and as PPIE co-lead for the NIHR GOSH BRC, she receives some salary support from the NIHR Biomedical Research Centre at GOSH. PL is also a Senior Centre Affiliate at The Centre for Adolescent Rheumatology Versus Arthritis at UCL, UCLH and GOSH, which is supported by Versus Arthritis (21593). All research at Great Ormond Street Hospital NHS Foundation Trust and UCL Great Ormond Street Institute of Child Health is made possible by the NIHR Great Ormond Street Hospital Biomedical Research Centre. The views expressed are those of the author(s) and not necessarily those of the NHS, the NIHR or the Department of Health.

## Appendix Supplementary materials

Image, application 1

Image, application 2

## Footnotes

Supplementary material associated with this article can be found in the online version at doi:10.1016/j.ero.2026.100258.
