# Supplementary material for: ‘Let’s work alongside it’: healthcare professionals’ perspectives of generative AI in supporting care at home in paediatric rheumatology
Source: EULAR Rheumatol Open. 2026 Oct 19;2(4):100258. doi: 10.1016/j.ero.2026.100258 (PMC13695108; doi:10.1016/j.ero.2026.100258)
Supplement: Supplementary file 1 [file mmc2.pdf]

Supplementary material S2

Welcome and Introduction

- Thank you for joining today's session!
- This discussion is part of a UCL MSc Research project in collaboration with the IMPACT team.
- We really value your insight as experienced healthcare professionals.

About this Study

- Exploring how healthcare professionals in paediatric rheumatology view the use of generative AI tools to support hands-on care tasks at home.
- These tools don't replace care, but could assist between appointments.

Format for this Session

- 30 minute group discussion
- Walk-through 3 scenarios
- After each one, we'll ask for your thoughts
- Everything is confidential and voluntary

Quick Introductions

- Please share
  - Your name
  - Your profession
  - Where you're based

Scenario 1:
A family is using a GenAI tool to assist with weekly methotrexate injections at home. They may feel uncertain, even if they've been shown how to do it.

- Plays a tailored instructional video
- Gives real-time, step-by-step voice guidance & visual references
- Prompts parent to take a photo of the injection site
- Uses visual recognition to check positioning and offer feedback
- Logs time, site, and comments for professional review

How It Works:

1. Uses reference models of ideal injection site locations
2. Applies landmark detection to recognize skin surface and anatomy
3. Compares image to a "safe zone" to assess accuracy (not too close to joint, bone, or nerve)

Scenario 2:
A parent is concerned their child may be experiencing a flare-up. They use a GenAI assistant to guide them through a self-check at home.

- Prompts the parent with step-by step voice and visuals cues to check specific joints and what they should be feeling for and how to assess
- Prompts parent to take video of palpation method/picture of flare-up site
- Checks positioning or technique accuracy
- Asks structured follow-up questions to what they are feeling and offers feedback
- Gives recommendations for next steps

How It Works:

1. Uses standard criteria and symptom patterns from clinical guidelines to detect flare-ups.
2. Refers to models showing correct self-check techniques and where flare-ups usually happen.
3. Gives advice based on how serious the symptoms are and medical guidelines.

Scenario 3:
A 10-year-old with JIA is prescribed daily range-of-motion exercises. At home, the family uses a GenAI tool to guide the child through their routine.

- Plays short videos showing correct exercise techniques
- Uses the camera to track movements
- Gives real-time feedback
- Offers encouragement and reminders to stay on track
- Allows the parent to log pain or challenges after each session
- Tracks adherence and progress over time

How It Works:

1. Uses pose estimation to monitor joint angles and movement quality
2. Compares the child's movement to preset reference patterns
3. Gives simple corrective prompts and motivational cues

Thank you!
